# Characteristics of self-management education and support programmes for people with chronic diseases delivered by primary care teams: a rapid review

**DOI:** 10.1186/s12875-024-02262-2

**Published:** 2024-01-31

**Authors:** Emmanuel Allory, Jordan Scheer, Vincent De Andrade, Ronan Garlantézec, Rémi Gagnayre

**Affiliations:** 1 Department of General Practice, Univ Rennes, 2 Av. du Professeur Léon Bernard, Rennes, 35000 France; 2 CHU Rennes, Inserm, CIC 1414 (Centre d’Investigation Clinique de Rennes), Rennes, 35000 France; 3 LEPS (Laboratoire d’Education Et Promotion en Santé), University of Sorbonne Paris Nord, Villetaneuse, UR 3412, F-93430 France; 4 CHU de Rennes, Univ Rennes, Inserm, EHESP (Ecole Des Hautes Etudes en Santé Publique), Irset - UMR_S 1085, Rennes, 35000 France

**Keywords:** Primary health care, Self-management, Patient education as topic, Organisational models, Chronic disease, Multimorbidity

## Abstract

**Background:**

Primary care actors can play a major role in developing and promoting access to Self-Management Education and Support (SMES) programmes for people with chronic disease. We reviewed studies on SMES programmes in primary care by focusing on the following dimensions: models of SMES programmes in primary care, SMES team’s composition, and participants’ characteristics.

**Methods:**

For this mixed-methods rapid review, we searched the PubMed and Cochrane Library databases to identify articles in English and French that assessed a SMES programme in primary care for four main chronic diseases (diabetes, cancer, cardiovascular disease and/or respiratory chronic disease) and published between 1 January 2013 and 31 December 2021. We excluded articles on non-original research and reviews. We evaluated the quality of the selected studies using the Mixed Methods Appraisal Tool. We reported the study results following the PRISMA guidelines.

**Results:**

We included 68 studies in the analysis. In 46/68 studies, a SMES model was described by focusing mainly on the organisational dimension (*n* = 24). The Chronic Care Model was the most used organisational model (*n* = 9). Only three studies described a multi-dimension model. In general, the SMES team was composed of two healthcare providers (mainly nurses), and partnerships with community actors were rarely reported. Participants were mainly patients with only one chronic disease. Only 20% of the described programmes took into account multimorbidity. Our rapid review focused on two databases and did not identify the SMES programme outcomes.

**Conclusions:**

Our findings highlight the limited implication of community actors and the infrequent inclusion of multimorbidity in the SMES programmes, despite the recommendations to develop a more interdisciplinary approach in SMES programmes. This rapid review identified areas of improvement for SMES programme development in primary care, especially the privileged place of nurses in their promotion.

**Trial registration:**

PROSPERO 2021 CRD42021268290.

**Supplementary Information:**

The online version contains supplementary material available at 10.1186/s12875-024-02262-2.

## Background

The number of people with chronic diseases has been rising worldwide [1], and one third of them has more than one chronic condition [2]. Several international scientific societies recommend Self-Management Education and Support (SMES) interventions because they can improve the quality of life of people with chronic conditions [3–5]. SMES programmes are defined as the provision of the foundation to help people manage their chronic disease, and guide their health-related decisions and activities [6]. However, several authors highlighted that attendance to SMES programmes by patients is low, despite their widely acknowledged benefits [7, 8]. Primary care actors can play a major role in developing SMES programmes and improving the patients’ access to these interventions [6, 9, 10].

However, to better meet the needs of people living with chronic diseases, the different dimensions of SMES interventions in primary care need to be reconsidered [11]. First, the healthcare organisation (HCO) is a major category to consider when developing a model of SMES delivery. In the *Chronic Care Model (CCM),* created by Wagner in 2001 and revisited in 2019, the delivery system design, decision support and clinical information system are included in the HCO [12]. However, in 2018, Reynolds showed in a systematic review that more evidence is needed about the impact of the SMES programme organisational dimension on professional and patient outcomes in primary care [13]. Second, several authors stated that SMES programmes should be based on the social cognitive theory, particularly the self-efficacy concept [14–16]. Yet, in 2019, a systematic review of randomised controlled trials found that none of the studied SMES programmes included a theoretical or conceptual framework [17]. Third, although the educative content of SMES programmes seems well established, the educational theory developed by the team to accompany patients in their skill and competence development is rarely described [15]. These three dimensions (organisational, social/behavioural, and educational), which can be combined or not by authors, must all be taken into account because the model of care can influence the results of SMES programmes [11].

Besides the models, the SMES team composition also needs to be considered. As underlined by the CCM model, collaboration among the primary care healthcare providers (HCP) is a major component of the model [12]. The emergence of new HCPs in primary care offers new workforce and the opportunity to rethink the SMES team collaboration [7, 18]. However, in 2022, the scoping review by Longhini et al. showed that the SMES team members’ roles and responsibilities in delivering care were not precisely described in studies on SMES [19]. In addition, the participation of community actors should be encouraged and strengthened. This will help the population to better identify the proposed SMES programme and the HCP team to better adapt the SMES activities to the population [20]. Yet, different studies showed the lack of collaboration between HCPs and community actors, especially social services [13, 19, 21].

Lastly, the profile of participants also should be taken into account in the SMES model due to the current primary care challenges, particularly multimorbidity. In 2014, Rijken et al. stressed that the development of a multimorbidity approach in SMES programmes is a priority [14, 22]. Due to the primary care teams’ key role in the management of people with multimorbidity, HCO must undergo a radical change [23, 24]. Indeed, the care of patients with multimorbidity is time-consuming and multimorbidity management might create difficulties among primary care providers [25, 26].

Previous studies highlighted a gap between the patients’ needs, due to the increasing number of people with chronic diseases, and the type of SMES interventions implemented in primary care. More data on the SMES model dimensions (organisational, social/behavioural and educational), SMES team composition and roles, and participants’ characteristics are needed.

The objective of this rapid review was to identify studies on SMES programmes for people with chronic diseases in primary care, with a specific focus on the different dimensions of the models, the SMES team composition, and the participants in order to highlight elements that need to be improved.

## Method

This rapid review was performed following the World Health Organisation practical guide for rapid reviews [27]. This approach was chosen due to the time and skills necessary to execute a systematic review in the context of the rapid increase of the volume of publications on SMES in primary care [13, 28]. This guide lists six different rapid review approaches with different feasibility, timeliness, comprehensiveness and quality assessment levels. For this rapid review, approach 6 was chosen because it focuses on comprehensiveness (i.e. wide consideration of the subject) and quality assessment (i.e. good measurement and evaluation of the quality of the selected studies). Therefore, our search strategy focused on more than one database, with date and language selection. Two independent reviewers (EA, JS) selected the articles, performed data abstraction and bias assessment using the Mixed Methods Appraisal Tool (MMAT) [27]. The PRISMA guidelines were followed for reporting the study results [29] (Additional files 1 and 2). The protocol of this rapid review was registered in PROSPERO and can be accessed at: https://www.crd.york.ac.uk/PROSPERO/display_record.php?RecordID=268290.

### Data sources and search strategy

Two databases were searched: PubMed and the Cochrane Database of Systematic Reviews. PubMed was chosen because it is the second source of health education publications and using other databases to identify studies on therapeutic interventions does not significantly change the search outcome [30, 31]. The Cochrane database was chosen because of its systematic approach for reviewing randomised controlled trials.

All authors and a documentalist (VDA) contributed to defining the following search strategy: only articles in English and French, and published from 1 January 2013 to 31 December 2021. The beginning date (1 January 2013) was chosen because following an international survey in 2014, it was recommended that SMES programmes in primary care should better address multimorbidity and that such programmes should be better integrated in the community [14].

The search strategy covered the following four domains: (1) primary care or primary healthcare; (2) models considered according to their organisational or educational dimension; (3) self-management under various names due to naming inconsistency in the literature [32]; in our article, self-management has been chosen as the main term, due to its focus on chronic disease, whereas self-care mainly expresses the ability of people to prevent a disease [15, 33]; and (4) the four most common chronic conditions: diabetes, cancer, cardiovascular disease, and respiratory chronic disease (chronic obstructive pulmonary disease and asthma) [1]. For both databases, the following string of keywords was used: ((‘primary health care’[MH] OR ‘primary health care’[TW] OR ‘primary care’[TW]) AND (‘organisational model’[TW] OR ‘models, organisational’[MH] OR ‘models, educational’[MH] OR ‘educational model’[TW] OR (‘organisat*’[TW] AND ‘model*’[TW]) OR (‘educ*’[TW] AND ‘model*’[TW]) OR (‘theor*’[TW] AND ‘model*’[TW])) AND (‘patient education as topic’[MH] OR ‘patient education’[TW] OR ‘patient teaching’ [TW] OR ‘self-care’[MH] OR ‘self-care’[TW] OR ‘self-management’[MH] OR ‘self-management’[TW] OR ‘health education’[MH] OR ‘health education’[TW] OR ‘health promotion’[MH] OR ‘health promotion’ [TW]) AND (‘non communicable diseases’ [MH] OR ‘chronic disease’ [MH] OR ‘multimorbidity’ [MH] OR ‘chronic*’[TW] OR ‘diabetes*’ [TW] OR ‘cancer’ [TW] OR ‘cardiovascular disease’ [TW] OR ‘asthma’ [TW] OR ‘chronic obstructive pulmonary disease’ [TW])).

### Study selection

Citations were downloaded and screened in Rayyan® (a web-based tool for evidence synthesis, https://www.rayyan.ai/). Two reviewers (EA, JS) independently screened titles and abstracts and checked the exclusion and inclusion criteria (see below). Conflicts were solved by discussion. When the two reviewers could not decide whether an article should be retained on the basis of its title and abstract, they screened the full text.

Inclusion criteria were:

- Studies on primary care, according to the definition by Starfield et al.: the first contact, realising continuity and coordination of care, and having a global and community approach [34].
- Studies on one of the four most prevalent chronic diseases, or on multimorbidity [1].
- Studies that evaluated a SMES intervention, as defined by the American Diabetes Association, using qualitative and/or quantitative methods [6].

Exclusion criteria were:

- Review articles.
- Articles that did not report results from original research, such as protocol studies, expert opinion articles and recommendations made by authors.
- Studies on routine care without a dedicated SMES intervention.

For each article that passed the initial screening, two reviewers (EA and JS) independently read the full text to determine whether it met the inclusion or exclusion criteria. In case of conflict between reviewers concerning the inclusion of a study, a third person brought his expertise (RGy).

### Data abstraction, quality assessment and analysis

Data abstraction from the selected articles was carried out by two authors (EA, JS) and then all authors analysed the included studies in four steps. First, the quality of the included studies was evaluated using the MMAT [35]. This scale allows assessing quantitative and qualitative methods in a mixed-methods approach (Additional file 3). Second, the model of each SMES programme was recorded, without predefined categories. RGz (professor in public health) particularly focused on the organisational models and RGy (professor in health education) focused on the social and health behaviour models and educational models. Third, the characteristics of the SMES team composition were extracted: number and occupation of each member, presence of a patient partner [36], community partner, and/or hospital participation. Fourth, the following data were extracted from the studies: participants’ characteristics (patient, caregiver), number of chronic diseases (one or multimorbidity), recruitment procedure, and programme size as defined in the primary care classification (micro level: < 50,000 habitants and/or < 5 primary care centres; meso level: > 50,000 habitants and/or ≥ 5 and < 10 primary care centres; and macro level: > 10 primary care centres) proposed by Jan De Maeseneer et al. [37].

All data were collected in an Excel file and shared among authors, in a blinded way.

## Results

### Study selection

**Fig. 1:**
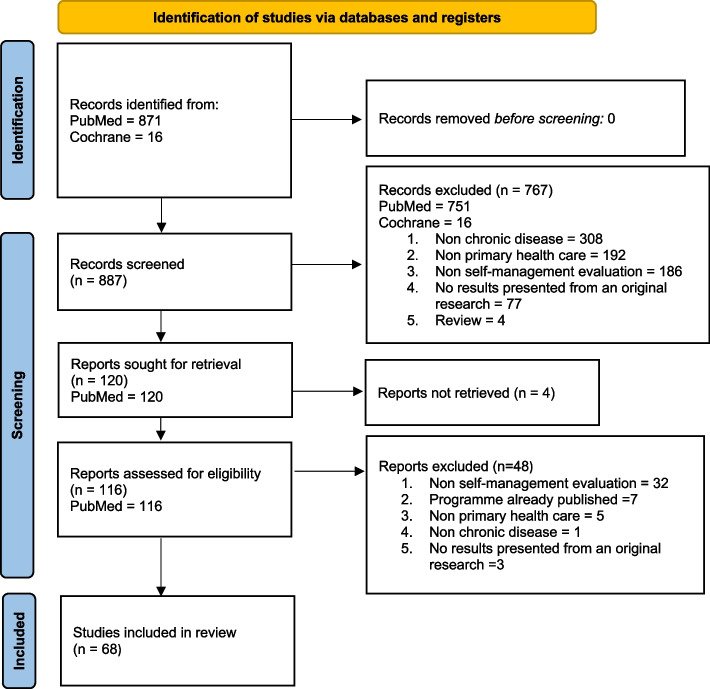
Prisma flow diagram

The initial search in the PubMed and Cochrane databases resulted in the inclusion of 887 studies, with no duplicate found (Fig. 1). After reading the title and abstract, 767 studies were excluded because they were not on chronic diseases (*n* = 308) or primary healthcare (*n* = 192), did not assess a SMES programme (*n* = 186), did not describe results from original research (*n* = 77), or were literature reviews (*n* = 4). This resulted in the selection of 120 articles, but only 116 articles were retained because the full text of four articles could not be obtained even after contacting the authors. After reading the full text of the 116 articles, 48 articles were excluded because they did not evaluate a SMES programme (*n* = 32), the same programme had already been evaluated in another article (*n* = 7), they did not concern primary healthcare (*n* = 5) or a chronic disease (*n* = 1), and they did not present results from original research (*n* = 3).

### Study characteristics and quality

Among the 68 selected studies, 36 were carried out in North America (United States *n* = 30, Canada *n* = 5, Mexico *n* = 1,), 12 in Europe (United Kingdom *n* = 3, Netherlands *n* = 3, Spain *n* = 2, Belgium *n* = 1, Denmark *n* = 1, Italy *n* = 1, Sweden *n* = 1), 11 in Asia (Hong Kong *n* = 3, Japan *n* = 2, China *n* = 1, Malaysia *n* = 1, Philippines *n* = 1, Thailand *n* = 1), 5 in Oceania (Australia *n* = 3, American Samoa *n* = 1, New Zealand *n* = 1), 2 in South America (Brazil *n* = 2), 1 in Central America (Guatemala *n* = 1), and 1 Africa (South Africa *n* = 1). Study design was variable: quantitative non-randomised study (*n* = 26), quantitative randomised control trial (*n* = 25), mixed methods study (*n* = 7), qualitative study (*n* = 6), quantitative descriptive study (*n* = 3), and cost-effectiveness analysis (*n* = 1). The mean MMAT score was 3.4 [min 0, max 5].

### SMES models, SMES teams, chronic condition(s), territory level

Table 1 provides an overview of the characteristics of the SMES models in the 68 studies retained for this rapid review. In 46 studies, a single-dimension model (*n* = 38) or multiple models (*n* = 8) were described. In the studies with multiple models, models were not combined in five studies, and were combined in a multi-dimension model in three studies. Among the 38 studies on a single-dimension model, 24 articles used an organisational model, 13 a social and health behaviour model, and 1 an educational model.

**Table 1:** Characteristics of the intervention models in the 68 studies

Number of models and integration	Dimension		Type/Name of the model	Authors	Articles
*Single-dimension model (n* = *38 studies)*	*Organisational model (n* = *24 studies)*		*Chronic Care Model (CCM)*	Wagner [38–40], Bodenheimer [41, 42], Coleman [43],	Boland 2015, Gucciardi 2015, Jiao 2015, Kane 2016, Ramli 2016, Francesconi 2019, Zupa 2019, Saude 2020, Vitale 2020
			*Added HCP (psychologist, certified diabetes educator, nurse)*		Brumisholz 2014, Grigg 2014, Edelman 2015
			*Teamlet model of primary care*	Bodenheimer [44]	Willard Grace 2015
			*Brisbane South Complex Diabetes Service (BSCDS)*	Jackson [45]	Hepworth 2013
			*Telemedicine for Reach, Education, Access, and Treatment (TREAT) model*	Toledo [46]	Siminerio 2014
			*Primary Care Medical Home*	American Association of Family Physicians [47]	Sepers 2015
			*Support nucleus for the family healthcare*	Brazilian Ministry of Health	Kuhmmer 2016,
			*Chronic Disease Self-Management Programme (CDSMP)*	Lorig [48]	Moreno 2018
			*Iora Health*	Iora [49]	Shah 2019
			*New Zealand specific DSME programme*	Developed by the authors	Krebs 2013
			*Project Impact*	Developed by the authors [50]	Bluml 2014
			*First line diabetes care (FiLDCare) Project*	Developed by the authors	Ku 2014
			*Care Management Medical Home Centre*	Developed by the authors	Page 2015
			*Guidance Research on Illness Perception(COPD-GRIP) intervention*	Developed by the authors	Weldam 2017
	*Social and health behaviour models (n* = *13 studies)*	*Behavioural model (n* = *10 studies)*	*Motivational approach (interview, model)*	Rollnick [51] Maindal [52]	Mash 2015, Maindal 2014 Contant 2019, Represas Carrera 2020
			*Transtheoretical model of behaviour change*	Prochaska [53, 54]	Liddy 2014, Coultas 2017, Moriyama 2021
			*Managing Illness by Empowerment of Self-care and Harmonisation of Patient and Practitioner Agendas (MESH)*	Developed by the authors	Denford 2013
			*Not specified*		Rovner 2020, Batch 2021
		*Social-cognitive model (n* = *3 studies)*	*Self-efficacy*	Bandura [55, 56]	Ryan 2013
			*Empowerment theory*	Funnell [57]	Piatt 2018
			*Common Sense model of sense-regulation*	Leventhal [58]	Van Puffelen 2019
	*Educational model (n* = *1 study)*		*Didactic model*	Berglund [59]	Kjellsdotter 2020
*Multiple models not combined (n* = *5 studies)*	*Organisational (CCM) and educational model (5A’s)*			Stellefson [60], Glasgow [61]	Talavera 2021
	*Motivational approach and self-efficacy*			Emmons [62], Bandura [63]	Eakin 2014
	*Motivational approach and health belief model*			Hayden [64], Hettema [65]	Ansari 2020
	*Health belief model and transtheoretical model of behaviour change*			Janz [66], Prochaska [67]	Fort 2015
	*Motivational approach, transtheoretical model of behaviour change, and educational model (5A’s)*			Miller [68], Goldstein [69]	Ruggiero 2014
*Multi-dimension model (n* = *3 studies)*	*Precede/Proceed model (behavioural, ecological, educational and organisational)*			Green [70]	DePue 2013
	*HelP Diabetes (behavioural, ecological and organisational)*			Corbin & Strauss [71]	Murray 2017
	*Medication Therapy Management model (behavioural and organisational)*			American Pharmacists Association [72]	Rodis 2017

*CCM* Chronic care model, *COPD* Chronic Obstructive Pulmonary Disease, *DSME* Diabetes self-management education, *HCP* Health Care Provider

CCM was the most frequently used organisational model (*n* = 9). Organisational models were developed by the authors in five studies. In three studies, the organisational model consisted in adding one HCP. In seven studies, a pre-existing organisational model was used. The chronic disease self-management programme was the only model with a community-based approach [48]. The Teamlet model of primary care included one clinician and two health coaches [44]. Two models were implemented at the primary care practice level: the primary care medical home model [47] and the Iora health model [49]. In the support nucleus for the family healthcare model by the Brazilian ministry of health, a multidisciplinary educational health care team was added to the primary care practice [73]. Two models were implemented in secondary care: the Brisbane South Complex Diabetes Service model (integrated community and specialist model) [45] and the Telemedicine for Reach, Education, Access, and Treatment model (video consultations with a diabetes specialist and a diabetes educator) [46].

Among the studies that chose a social and health behaviour model (*n* = 13), ten used a behavioural model and three a social cognitive model. The most frequent behavioural model was the motivational approach [51], followed by the transtheoretical model of behaviour change [53]. One behavioural model was developed by the authors [74]. The three studies on social cognitive models used the self-efficacy model described by Bandura [55], the empowerment theory developed by Funnell [57], and the common sense model of sense-regulation [58].

Only one study described an educational model created by the authors [59].

The five studies with non-combined models used mostly social and health behaviour models. Three studies evaluated a multi-dimension model.

In the 68 studies, the mean number of HCPs in the SMES team was 2 (range: 1–7) (Table 2). They were mainly nurses (*n* = 36 studies), followed by dieticians (*n* = 17 studies), general practitioners (GP) (*n* = 13 studies), qualified peers (*n* = 12 studies), community health workers (*n* = 6 studies), peer leaders (*n* = 3 studies), health promoters (*n* = 2 studies), patient navigators (*n* = 1 study), and educators (*n* = 9 studies). Other HCP types were part of the SMES team in 13 studies: physiotherapists (*n* = 4), physical educators (*n* = 2), respiratory therapists (*n* = 2), podiatrists (*n* = 2), occupational therapists (*n* = 1), smoking cessation therapists (*n* = 1), and optometrists (*n* = 1). Pharmacists were included in 7 studies, health coaches in 6, medical assistants in 5 (including health technicians), and social workers in 3. Besides GPs, other physicians were included in the SMES team: endocrinologist (*n* = 1 study), health officer (*n* = 1 study), medical officer (*n* = 1 study), and a specialist without further information (*n* = 1 study). Health students also were involved in the SMES intervention (*n* = 2 studies). One study proposed the notion of primary care team. Two studies did not give any information on the HCP number and type. A partnership with a community actor was described in 17/68 studies. Some studies reported the implication of the hospital (*n* = 7 studies), of the patient as partner (*n* = 5 studies), and of community partners (*n* = 5 studies; lay community workers, community champion, patient association, advisory panel with community members, community leaders, and village health volunteers).

**Table 2:** Characteristics of the selected studies, SMES team composition, and participants in the programme

Study				SMES team composition			Participants		
**Authors (year)**	**Country (ref)**	**Type**	**MMAT**	**Number**	**HCP**	**Community partner**	**Participant (Disease)**	**Recruitment**	**Territory level**
Adachi et al. (2013)	Japan [75]	QRCT	5	1	Dietician	None	Patient (Diabetes)	PCP	Meso
Denford et al.(2013)	United-Kingdom [74]	Ql	5	1	Nurse	None	Patient (Asthma)	HDB	Micro
De Pue et al. (2013)	American Samoa [76]	QRT	5	2	Nurse, CHW	Hospital	Patient (Diabetes)	HDB	Micro
Hepworth et al. (2013)	Australia [77]	Ql	3	4	Endocrinologist, GP, educator, podiatrist	Hospital	Patient (Diabetes)	HDB	Micro
Krebs et al. (2013)	New Zealand [78]	QNR	2	2	Dietician, nurse	Hospital, community partners (lay community workers)	Patient (Diabetes) and caregiver	PCP, diabetes organisation and secondary care	Macro
Ryan et al. (2013)	United States [79]	QNR	3	2	Nurse, dietician	None	Patient (Diabetes)	PCP	Micro
Shaw et al. (2013)	United States [80]	MM	4	1	Nurse	None	Patient (Hypertension)	HDB	Macro
Bluml et al. (2014)	United States [81]	QNR	3	2	Pharmacist, PCT	Patient partner, community partner (community champion)	Patient (Diabetes)	NA	Macro
Brunisholz et al. (2014)	United States [82]	QNR	3	2	Nurse, dietician	None	Patient (Diabetes)	HDB	Macro
Eakin et al. (2014)	Australia [83]	QRCT	4	1	NA	None	Patient (Diabetes)	HDB, PCP	Meso
Grigg et al. (2014)	United States [84]	QNR	1	1	Educator	None	Patient (Diabetes)	HDB	Meso
Ku et al. (2014)	Philippines [85]	QNR	4	4	Health officer, nurse, CHW, midwife	None	Patient (Diabetes)	PCP	Meso
Liddy et al. (2014)	Canada [86]	Ql	5	1	Health coach	None	Patient (Diabetes)	PCP	Meso
Maindal et al. (2014)	Denmark [87]	QRCT	4	4	Nurse, GP, dietician, physiotherapist	None	Patient (Diabetes)	Participant to a national programme	Meso
Ruggiero et al. (2014)	United States [88]	QRCT	4	1	Medical assistant	None	Patient (Diabetes)	PCP	Meso
Siminerio et al. (2014)	United States [89]	QNR	2	2	Specialised doctor, nurse	Hospital	Patient (Diabetes)	PCP	Micro
Thom et al. (2014)	United-States [90]	QRCT	4	1	Health coach	None	Patient^a^ (Diabetes, Hypertension, Dyslipidaemia)	HDB	Meso
Boland et al. (2015)	The Netherlands [91]	MM	0	4	GP, practice nurse, physiotherapist, dietician	Hospital	Patient (COPD)	NA	Meso
Cowden et al. (2015)	United States [92]	QRCT	3	1	GP	None	Patient (Asthma) and caregiver	HDB, PCP	Meso
Edelman et al. (2015)	United States [93]	QRCT	5	1	Nurse	None	Patient^a^ (Diabetes, Hypertension)	HDB, PCP	Meso
Fort et al. (2015)	Guatemala [94]	QNR	4	4	Health promoter, nurse, dietician, GP,	None	Patient^a^ (Diabetes, Hypertension)	HDB	Meso
Gucciardi et al. (2015)	Canada [95]	Ql	5	2	Dietician, nurse	None	Patient (Diabetes)	PCP	Meso
Jiao et al. (2015)	Hong Kong [96]	QNR	4	7	GP, nurse, practice nurse, podiatrist, dietician, physiotherapist, optometrist	None	Patient (Diabetes)	NA	Macro
Mash et al. (2015)	South Africa [97]	QRCT	0	1	Health promoters	None	Patient (Diabetes)	NA	Meso
Mino-Leon et al. (2015)	Mexico [98]	QNR	2	2	Pharmacist, nurse	None	Patient^a^ (Diabetes, Hypertension)	PCP	Meso
Page et al. (2015)	United States [99]	QD	5	2	Nurse, health technician	None	Patient (Diabetes)	HDB	Meso
Sepers et al. (2015)	United States [100]	MM	2	5	Educator, health coach, medical assistant, CHW, nurse	Patient partner	Patient (Diabetes)	NA	Meso
Willard-Grace et al. (2015)	United States [101]	QRCT	5	1	Medical assistant	None	Patient^a^ (Diabetes, Hypertension, Dyslipidaemia)	HDB, PCP	Micro
Wong et al. (2015)	Hong Kong [102]	QNR	4	3	Dietician, nurse, social worker	None	Patient (Diabetes)	Participant to a DSME programme	Macro
Zhong et al. (2015)	China [103]	MM	1	2	Peer leader, nurse	Patient partner	Patient (Diabetes)	HDB, PCP	Meso
Chomko et al. (2016)	United States [104]	QNR	3	3	Nurse, dietician, social worker	None	Patient (Diabetes)	NA	Meso
Kane et al. (2016)	United States [105]	QNR	5	1	CHW	Hospital	Patient (Diabetes)	PCP	Meso
Kuhmmer et al. (2016)	Brazil [73]	QRCT	4	6	Physical educator, pharmacist, dietician, GP, nurse, social worker	None	Patient (Hypertension)	PCP	Micro
Loskutova et al. (2016)	United States [106]	Mm	2	1	Patient navigator	Community partner (patient association)	Patient (Diabetes)	PCP	Meso
Odnoletkova et al. (2016)	Belgium [107]	CEA	NA	1	Nurse	None	Patient (Diabetes)	Health insurance fund	Macro
Ramli et al. (2016)	Malaysia [108]	QRCT	5	6	GP, medical officer, medical assistant, dietician, pharmacist	Community partners (advisory panel with community members)	Patient (Diabetes)	Investigators in waiting area of practices	Meso
Murray et al. (2017)	United Kingdom [109]	QRCT	5	1	Nurse	None	Patient (Diabetes)	HDB, PCP	Macro
Rodis et al. (2017)	Unites States [110]	QD	1	1	Pharmacist	None	Patient^a^ (Diabetes, Hypertension)	HDB	Meso
Weldam et al. (2017)	The Netherlands [111]	QRCT	4	1	Practice nurse	None	Patient (COPD)	PCP	Macro
Yeung et al. (2017)	United States [112]	QNR	5	1	Pharmacist	None	Patient^a^ (CHF, Diabetes, Hypertension)	PCP	Meso
Benedict et al. (2018)	United States [113]	QNR	4	1	Pharmacist	None	Patient (Diabetes)	HDB, PCP	Micro
Bourbeau et al. (2018)	Canada [114]	QNR	4	1	Respiratory therapist	None	Patient (COPD)	PCP	Meso
Coultas et al. (2018)	United States [115]	QRCT	2	1	Health coach	None	Patient (COPD)	NA	Micro
Piatt et al. (2018)	United States [116]	QRCT	4	2	Educator, peer leader	Patient partner	Patient (Diabetes)	NA	Meso
Torres et al. (2018)	Brazil [117]	QRCT	3	3	Nurse, dietician, physiotherapist	None	Patient (Diabetes)	HDB	Meso
Aekplakorn et al. (2019)	Thailand [118]	QRCT	3	NA	NA	Community partner (community leaders, village health volunteers)	Patient (Prediabetes)	Screening programme	Macro
Contant et al. (2019)	Canada [119]	QRCT	4	5	Educator, dietician, physical educator, respiratory therapist, smoking cessation therapist	None	Patient^a^ (Asthma, Cardiovascular disease, COPD, Diabetes and Prediabetes, Dyslipidaemia, Obesity, Smoking)	PCP	Meso
Francesconi et al. (2019)	Italy [120]	QNR	2	2	GP, nurse	None	Patient (CHF)	PCP	Macro
Moreno et al. (2019)	Spain [121]	QRCT	4	2	Peer leader, educator	Patient partner	Patient (Diabetes)	PCP	Macro
Shah et al. (2019)	United Kingdom [122]	QD	4	1	Health coach	None	Patient^a^ (NA)	NA	Macro
Siminerio et al. (2019)	United States [123]	Ql	4	1	Educator	None	Patient (Diabetes)	NA	Meso
Srulovici et al. (2019)	Israel [124]	QNR	3	1	Nurse	None	Patient (Diabetes) caregiver	PCP	Macro
Van Puffelen et al. (2019)	The Netherlands [125]	MM	1	1	Psychologist	None	Patient (Diabetes), caregiver	PCP	Micro
Zupa et al. (2019)	United States [126]	QNR	3	1	Educator	None	Patient (Diabetes)	PCP	NA
Ansari et al. (2020)	Australia [127]	QNR	2	1	Practice nurse	None	Patient^a^ (Diabetes + another not specified chronic disease)	HDB	Macro
Kjellsdotter et al. (2020)	Sweden [128]	Ql	4	1	Diabetes nurse	None	Patient (Diabetes)	NA	Micro
Mammen et al. (2020)	United States [129]	MM	3	1	Nurse	None	Patient (Asthma)	Research assistant	Micro
Rovner et al. (2020)	United States [130]	QRCT	4	1	Occupational therapist	None	Patient^a^ (Diabetes, Mild cognitive impairment)	HDB, research assistant	Micro
Saude et al. (2020)	United States [131]	QNR	2	2	Family nurse practitioner, nursing student	None	Patient^a^ (Diabetes, Hypertension)	NA	Micro
Vitale et al. (2020)	Canada [132]	QNR	4	2	Nurse, dietician	None	Patient (Diabetes)	PCP	Macro
Willard-Grace et al. (2020)	United States [133]	QRCT	3	1	Health coach	Hospital	Patient (COPD)	HDB, PCP, research assistant	Meso
Alibrahim et al. (2020)	Kuwait [134]	QNR	4	1	Educator	None	Patient (Diabetes)	PCP	Micro
Batch et al. (2021)	United States [135]	QNR	2	NA	NA	Hospital	Patient (Diabetes)	HDB, PCP	Macro
Represas Carre et al. (2021)	Spain [136]	QRCT	3	2	GP, nurse	Community partners (no indication)	Patient (Diabetes)	PCP, phone, waiting room with questionnaire and poster	Macro
Talavera et al. (2021)	United States [137]	QRCT	3	2	CHW, GP	None	Patient (Diabetes)	HDB, PCP	Micro
Moriyama et al. (2021)	Japan [138]	QNR	3	1	Nurse	None	Patient^a^ (Diabetes, Dyslipidaemia, Hypertension, Nephropathy)	HDB, PCP	Micro
Fu et al. (2021)	Hong Kong [139]	QRCT	2	2	GP, nurse	None	Patient (Hypertension)	PCP	Meso
Veldheer et al. (2021)	United States [140]	QNR	4	4	CHW, dietician, GP, medical resident	None	Patient^a^ (Diabetes, Obesity)	HDB, PCP	Micro

*CEA* Cost-effectiveness analysis, *CHF* Chronic heart failure, *CHW* Community health worker, *COPD* Chronic obstructive pulmonary disease, *GP* General practitioner, *HCP* Healthcare provider, *HDB* Health database, *MM* Mixed methods, *MMAT* Mixed methods of appraisal tool, *NA* Not available, *PCP* Primary care provider, *PCT* Primary care team, *QD* Quantitative descriptive, *Ql* Qualitative, *QNR* Quantitative non randomised, *QRCT* Quantitative randomised controlled trial

^a^Indicates SMES programme targeting people with multimorbidity

In 64 studies, the SMES programme was accessible only to the patients and in four studies to both caregivers and patients. The programme mainly focused on one chronic condition (*n* = 54): diabetes and prediabetes (*n* = 42), chronic obstructive pulmonary disease (COPD) (*n* = 5), asthma (*n* = 3), hypertension (*n* = 3), and chronic heart failure (*n* = 1). Among the 14 studies on multimorbidity, the main topics were diabetes and hypertension (*n* = 6), followed by diabetes, hypertension and dyslipidaemia (*n* = 1), diabetes, hypertension, dyslipidaemia and nephropathy (*n* = 1), diabetes and obesity (*n* = 1), diabetes and mild cognitive impairment (*n* = 1), heart failure, hypertension and diabetes medication (*n* = 1), diabetes, cardiovascular disease, COPD, asthma, tobacco, obesity, dyslipidaemia and prediabetes (*n* = 1), and COPD and another chronic disease (*n* = 1). One study did not specify the topic. Participants were recruited mainly by the primary care provider (*n* = 23), by invitation sent to patients identified by searching a health database (*n* = 12), and by the primary care provider plus identification by health database search (*n* = 11). The recruitment method was not indicated in twelve studies. In the other ten studies, the recruitment was through secondary care providers, another SMES programme, health insurance, recruitment by investigators or research assistants in the doctor’s waiting area. SMES programmes were mainly at the meso level (*n* = 31), followed by the micro and macro levels (*n* = 18 for each). One study did not describe the territory level of the SMES programme (Table 2).

## Discussion

### Main findings

In this rapid review on SMES programmes in primary care, we collected data on the model dimensions, SMES team composition, and participants. Most studies that referred to a model used a single-dimension organisational model, mainly the CCM. Only three studies described multi-dimension models. In general, the SMES team included two HCPs, mainly nurses. Partnerships with community actors were rarely described. Participants in the programme were mainly patients with one chronic disease. Only 20% of programmes considered multimorbidity.

### Comparison with the existing literature

SMES programmes are complex interventions in which several aspects of the healthcare system, HCPs and patients must be taken into account [17]. Our rapid review showed that in order of importance, HCPs consider first the organisational dimension of the SMES practice, and then the learning theory on which the SMES intervention is based. The major place of organisational models indicates that HCPs’ priorities are to better integrate the SMES programme in their daily practice and to take into account their own organisation. Although progress has been made, primary care teams still need to think how to deliver SMES programmes within their organisation [7]. Concerning learning theory-based models, most of them originated from the health and social psychology fields and fewer from the pedagogy field. This lack of educational models underlines the fact that most of the models described in the selected studies focused on understanding and explaining the participants’ behaviour, and not on supporting knowledge acquisition by the participants. This finding may be explained by the fact that for many years, psychology has been an integral part of medical training and is well integrated in the GPs’ practice [141]. Another hypothesis, as underlined by Lorig and Halman, is that this may express a different understanding of what SMES is by the SMES programme developers [142]. Lastly, only three studies included in this review used a multi-dimensional model to structure the SMES programme, by integrating the behavioural, ecological, educational and organisational dimensions. To better integrate SMES programmes in the healthcare system, a multi-dimensional model that takes into account the perspectives of different disciplines is needed. However, this more interdisciplinary approach has not been properly developed and tested yet. The precede-proceed model and the expanded CCM are two examples of multi-dimensional models for SMES implementation that could be considered [20, 70].

Nurses (family nurses, practice nurses, and diabetes nurses) were the main HCP involved in the different SMES programmes. Barreto et al. showed that although all HCPs in the team feel involved in the SMES intervention, nurses are seen by the team as important educators [67]. Similarly, Siminerio et al. reported in a qualitative study that both physicians and nurses agreed that nurses have a better understanding of psychosocial issues and are more likely than physicians to support patients in implementing the SMES programme [143]. These qualitative results were confirmed in the systematic review by Renders et al. showing that a greater involvement of nurses in diabetes management has positive effects on the patients’ outcome [9]. These findings demonstrate the importance of nurses in SMES and the place given to them by the SMES team and healthcare system. In agreement with literature data, we identified very few partnerships with community actors. In their systematic review on SMES, Reynolds et al. found that community resources were implicated in only 0.6% of the included studies [13]. Therefore, the recommendation by Barr et al. in 2003 to enhance community participation is still unmet [20]. This lack of partnerships with community actors in SMES programmes may suggest that HCPs have difficulties in taking ownership of health promotion principles. In 2017, a qualitative study showed that the successful implementation of health promotion principles by primary care providers is influenced by three dimensions: context, implementation process, and collaborative model [144]. Therefore, HCPs should find ways of promoting the integration of community actors in SMES programmes, possibly by integrating health promotion principles in the multidimensional model.

In agreement with the literature, our rapid review showed that SMES interventions in primary care often do not take into account multimorbidity. This is a worrying finding [2, 17]. Some results from previous studies support and promote the development of a multimorbidity approach in primary care. Cameron et al. showed that a moderate-to-severe comorbidity index was the strongest predictor of better self-management (especially maintenance behaviours) by patients [145]. The authors partly explained this result by suggesting that such patients had time to develop skills to cope with their first chronic disease and used this experience for the second chronic condition. Moreover, a multimorbidity model can help HCPs to provide better care. A qualitative study showed how GPs who develop a multimorbidity-focused SMES programme in their practice perceive the benefits of this approach in their care of people with multimorbidity [146]. The necessary collaboration among HCPs for taking care of people with multimorbidity can be facilitated by SMES interventions. However, some difficulties remain in the management of people with multimorbidity. In their systematic review of qualitative studies, Sinnott et al. showed that such difficulties can be classified in four areas: healthcare disorganisation and fragmentation, inadequacy of guidelines and evidence-based medicine, challenges in delivering patient-centred care, and barriers to shared decision making [63]. They also found that for implementing multimorbidity-focused SMES programmes, all healthcare system actors must be implicated and research on multimorbidity must be developed.

## Strengths and limitations

Our study has some limitations. First, our rapid review choose to focus only on two databases, although PubMed is one of the main source of publications on health education [30]. Scopus and CINAHL, which also are main databases of articles on health education, were not considered. This choice was necessary due to the rapid increase of publications on SMES interventions for chronic diseases in primary care [13]. This allowed us to thoroughly review the selected papers, despite our small team and time constraints. Second, this rapid review focused on three different aspects of SMES programmes: the model considered by the team, the team performing the SMES programme, and the participants in the programme. Each aspect could have been considered separately, but we wanted to use a global approach. Indeed, many studies showed that a successful SMES programme needs to be thought at multiple levels: health system organisation (HCPs and community partners), patient-clinician interaction (guided by the programme psychological and educational theory), and environmental support (caregivers’ integration) [147]. Third, this review did not focus on the outcomes of the selected studies (biomedical, pedagogical, psychosocial). As SMES programmes are recommended by the main learned societies of chronic disease, we considered that our main objective was to identify models of SMES in primary care [4]. Therefore, we focused on the model of care because according to Kumah et al., it may influence the effects of the SMES programme [11].

One of the strengths of this rapid review is that it brought together researchers with different expertise (education, public health, and primary care). In 2017, Mills et al. identified seven strategic directions that were described in the international chronic condition self-management support framework [148]. One of them was to work with stakeholders from different disciplines for developing programmes, as done by the research team that performed this rapid review.

## Conclusion

The increasing number of people with chronic diseases and with multimorbidity stresses the importance of SMES programmes, especially in primary care close to where patients live. Multidimensional models need to be promoted in which nurses and the partnership of community actors play major roles. Better integration of healthcare promotion principles also seems essential to ensure that SMES programmes are better integrated in the community.

### Supplementary Information

**Additional file 1. **PRISMA 2020 checklist.

**Additional file 2. **PRISMA 2020 for Abstracts Checklist.

**Additional file 3. **Results of the Mixed Methods Appraisal Tools (MMAT) evaluation (1).

## Data Availability

The dataset used and analysed in the current study is available from the corresponding author on reasonable request.

## Abbreviations

CCM: Chronic care model
CEA: Cost-effectiveness analysis
CHF: Cardiac heart failure
CHW: Community health workers
COPD: Chronic obstructive pulmonary disease
DSME: Diabetes self-management education
GP: General practitioner
HCP: Health care provider
HDB: Health database
MM: Multimorbidity
Mm: Mixed method
MMAT: Mixed methods appraisal tools
NA: Non-applicable
OECD: Organisation for economic co-operation and development
PC: Primary care
PCP: Primary care provider
PCT: Primary care team
PRISMA: Preferred reporting items for systematic reviews and meta-analyses
QD: Quantitative descriptive
Ql: Qualitative
QNR: Quantitative non randomised
QRCT: Quantitative randomised controlled trials
RR: Rapid review
SM: Self-management
SMES: Self-Management Education and Support
