# Supplementary material for: Characteristics of self-management education and support programmes for people with chronic diseases delivered by primary care teams: a rapid review
Source: BMC Prim Care. 2024 Jan 31;25:46. doi: 10.1186/s12875-024-02262-2 (PMC10829293; doi:10.1186/s12875-024-02262-2)
Supplement: Supplementary file 3 — Additional file 3. Results of the Mixed Methods Appraisal Tools (MMAT) evaluation (1). [file 12875_2024_2262_MOESM3_ESM.docx]

Additional file 3: Results of the Mixed Methods Appraisal Tools (MMAT) evaluation (1)

| **Title of the study** | **Name of the 1st author** | **Year** | **Type of study** | **Total quality appraisal** | **1** | **2** | **3** | **4** | **5** |
| --- | --- | --- | --- | --- | --- | --- | --- | --- | --- |
| Effects of lifestyle education program for type 2 diabetes patients in clinics: a cluster randomized controlled trial. | Adachi | 2013 | Quantitative randomised controlled trial | 5 | Yes | Yes | Yes | Yes | Yes |
| Processes of Change in an asthma Self-care intervention | Denford | 2013 | Qualitative | 5 | Yes | Yes | Yes | Yes | Yes |
| Nurse-community health worker team improves diabetes care in American Samoa: results of a randomized controlled trial. | De Pue | 2013 | Quantitative randomised controlled trial | 5 | Yes | Yes | Yes | Yes | Yes |
| Working with the team’: an exploratory study of improved type 2 diabetes management in a new model of integrated primary/secondary care | Hepworth | 2013 | Qualitative | 5 | Yes | Yes | Yes | Yes | Yes |
| A structured, group-based diabetes self-management education (DSME) programme for people, families and whanau with type 2 diabetes (T2DM) in New Zealand: An observational study. | Krebs | 2013 | Quantitative not randomised | 2 | No | Yes | No | No | Yes |
| Short and Long-Term Outcomes from a Multisession Diabetes Education Program Targeting Low-Income Minority Patients: A Six-Month Follow Up | Ryan | 2013 | Quantitative not randomised | 3 | No | Yes | Yes | No | Yes |
| Organizational factors associated with readiness to implement and translate a primary care based telemedicine behavioral program to improve blood pressure control: the HTN-IMPROVE study | Shaw | 2013 | Mixed methods | 4 | No | Yes | Yes | Yes | Yes |
| Improving outcomes for diverse populations disproportionately affected by diabetes: Final results of Project IMPACT: Diabetes | Bluml | 2014 | Quantitative not randomised | 3 | Yes | Yes | No | No | Yes |
| Diabetes self-management education improves quality of care and clinical outcomes determined by a diabetes bundle measure | Brunisholz | 2014 | Quantitative not randomised | 3 | No | Yes | Yes | No | Yes |
| Living Well With Diabetes: 24-Month Outcomes From a Randomized Trial of Telephone- Delivered Weight Loss and Physical Activity Intervention to Improve Glycemic Control | Eakin | 2014 | Quantitative randomised controlled trial | 4 | Yes | No | Yes | Yes | Yes |
| The Impact of Certified Diabetes Educators on Diabetes Performance and Variation Among Primary Care Sites Within an Integrated Health System | Grigg | 2014 | Quantitative not randomised | 1 | No | Yes | Can't tell | No | Can't tell |
| Effects of the First Line Diabetes Care (FiLDCare) self-management education and support project on knowledge, attitudes, perceptions, self-management practices and glycaemic control: a quasi-experimental study conducted in the Northern Philippines | Ku | 2014 | Quantitative not randomised | 4 | No | Yes | Yes | Yes | Yes |
| Health coaching in primary care: a feasibility model for diabetes care | Liddy | 2014 | Qualitative | 5 | Yes | Yes | Yes | Yes | Yes |
| Effect of a participant-driven health education programme in primary care for people with hyperglycaemia detected by screening: 3-year results from the Ready to Act randomized controlled trial (nested within the ADDITION-Denmark study) | Maindal | 2014 | Quantitative randomised controlled trial | 4 | Yes | Yes | No | Yes | Yes |
| Medical assistant coaching to support diabetes self-care among low-income racial/ethnic minority populations: Randomized controlled trial | Ruggiero | 2014 | Quantitative randomised controlled trial | 4 | Yes | Yes | No | Yes | Yes |
| Telemedicine for Reach, Education, Access, and Treatment (TREAT). Linking Telemedicine With Diabetes Self-management Education to Improve Care in Rural Communities | Siminerio | 2014 | Quantitative not randomised | 2 | No | No | Yes | No | Yes |
| Does health coaching change patients’ trust in their primary care provider? | Thom | 2014 | Quantitative randomised controlled trial | 4 | Yes | Yes | Yes | No | Yes |
| Exploring the variation in implementation of a COPD disease management programme and its impact on health outcomes: a post hoc analysis of the RECODE cluster randomised trial | Boland | 2015 | Mixed methods | 0 | No | No | No | No | No |
| The talking card: Randomized controlled trial of a novel audio-recording tool for asthma control | Cowden | 2015 | Quantitative randomised controlled trial | 3 | Yes | Can't tell | Yes | No | Yes |
| Nurse-Led Behavioral Management of Diabetes and Hypertension in Community Practices: A Randomized Trial | Edelman | 2015 | Quantitative randomised controlled trial | 5 | Yes | Yes | Yes | Yes | Yes |
| Impact evaluation of a healthy lifestyle intervention to reduce cardiovascular disease risk in health centers in San José, Costa Rica and Chiapas, Mexico | Fort | 2015 | Quantitative not randomised | 4 | Yes | Yes | Yes | Yes | Yes |
| Implementing Specialized Diabetes Teams in Primary Care in Southern Ontario | Gucciardi | 2015 | Qualitative | 5 | Yes | Yes | Yes | Yes | Yes |
| Long‑term effects of the multidisciplinary risk assessment and management program for patients with diabetes mellitus (RAMP‑DM): a population‑based cohort study | Jiao | 2015 | Quantitative not randomised | 4 | Yes | Yes | Yes | Yes | Can't tell |
| Cost-effectiveness of a diabetes group education program delivered by health promoters with a guiding style in underserved communities in Cape Town, South Africa | Mash | 2015 | Quantitative randomised controlled trial | 0 | Can't tell | Can't tell | Can't tell | Can't tell | Can't tell |
| Effectiveness of involving pharmacists in the process of ambulatory health care to improve drug treatment adherence and disease control | Mino-Leon | 2015 | Quantitative not randomised | 2 | Can't tell | Yes | No | No | Yes |
| Care Management Medical Home Center Model: Preliminary Results of a Patient-Centered Approach to Improving Care Quality for Diabetic Patients | Page | 2015 | Quantitative descriptive | 5 | Yes | Yes | Yes | Yes | Yes |
| Measuring the Implementation and Effects of a Coordinated Care Model Featuring Diabetes Self-management Education Within Four Patient-Centered Medical Homes | Sepers | 2015 | Mixed methods | 2 | Yes | No | Yes | No | No |
| Health Coaching by Medical Assistants to Improve Control of Diabetes, Hypertension, and Hyperlipidemia in Low-Income Patients: A Randomized Controlled Trial | Willard-Grace | 2015 | Quantitative randomised controlled trial | 5 | Yes | Yes | Yes | Yes | Yes |
| Increased number of structured diabetes education attendance was not associated with the improvement in patient-reported health-related quality of life: results from Patient Empowerment Programme (PEP) | Wong | 2015 | Quantitative not randomised | 4 | Yes | Yes | No | Yes | Yes |
| Peer Support for Diabetes Management in Primary Care and Community Settings in Anhui Province, China | Zhong | 2015 | Mixed methods | 1 | No | Yes | No | No | No |
| Enhancing Access to Diabetes Self-management Education in Primary Care | Chomko | 2016 | Quantitative not randomised | 3 | Yes | Yes | No | No | Yes |
| Improving diabetes care and outcomes with community health workers | Kane | 2016 | Quantitative not randomised | 5 | Yes | Yes | Yes | Yes | Yes |
| Effectiveness of multidisciplinary intervention on blood pressure control in primary health care: a randomized clinical trial | Kuhmmer | 2016 | Quantitative randomised controlled trial | 4 | Yes | Yes | No | Yes | Yes |
| Patient Navigators Connecting Patients to Community Resources to Improve Diabetes Outcomes | Loskutova | 2016 | Mixed methods | 2 | No | No | Yes | Yes | No |
| Delivering Diabetes Education through Nurse-Led Telecoaching. Cost-Effectiveness Analysis | Odnoletkova | 2016 | Cost-effectiveness analysis |  |  |  |  |  |  |
| Effectiveness of the EMPOWER-PAR Intervention in Improving Clinical Outcomes of Type 2 Diabetes Mellitus in Primary Care: A Pragmatic Cluster Randomised Controlled Trial | Ramli | 2016 | Quantitative randomised controlled trial | 5 | Yes | Yes | No | Yes | YEs |
| Web-based self-management support for people with type 2 diabetes (HeLP Diabetes): randomised controlled trial in English primary care | Murray | 2017 | Quantitative randomised controlled trial | 5 | Yes | Yes | Yes | Yes | Yes |
| Improving Chronic Disease Outcomes Through Medication Therapy Management in Federally Qualified Health Centers | Rodis | 2017 | Quantitative descriptive | 1 | Yes | Can't tell | No | No | No |
| The effectiveness of a nurse-led illness perception intervention in COPD patients: a cluster randomised trial in primary care | Weldam | 2017 | Quantitative randomised controlled trial | 4 | Yes | Yes | Yes | Yes | No |
| Low–health literacy flashcards & mobile video reinforcement to improve medication adherence in patients on oral diabetes, heart failure, and hypertension medications | Yeung | 2017 | Quantitative not randomised | 5 | Yes | YEs | Yes | Yes | Yes |
| Evaluation of a Pharmacist-Managed Diabetes Program in a Primary Care Setting Within an Integrated Health Care System | Benedict | 2018 | Quantitative not randomised | 4 | Yes | Yes | Yes | No | Yes |
| The Quebec Respiratory Health Education Network: Integrating a model of self-management education in COPD primary care | Bourbeau | 2018 | Quantitative not randomised | 4 | Yes | Yes | Can't tell | No | Yes |
| Home-based Physical Activity Coaching, Physical Activity, and Health Care Utilization in Chronic Obstructive Pulmonary Disease Chronic Obstructive Pulmonary Disease Self-Management Activation Research Trial Secondary Outcomes | Coultas | 2018 | Quantitative randomised controlled trial | 2 | Yes | No | No | No | Yes |
| Integration and Utilization of Peer Leaders for Diabetes Self-Management Support Results From Project SEED (Support, Education, and Evaluation in Diabetes) | Piatt | 2018 | Quantitative randomised controlled trial | 4 | Yes | Yes | Yes | No | Yes |
| Evaluation of the effects of a diabetes educational program: a randomized clinical trial | Torres | 2018 | Quantitative randomised controlled trial | 3 | Yes | No | No | Yes | Yes |
| Evaluation of a Community-Based Diabetes Prevention Program in Thailand: A Cluster Randomized Controlled Trial | Aekplakorn | 2019 | Quantitative randomised controlled trial | 3 | Yes | No | Yes | Yes | No |
| A multidisciplinary self-management intervention among patients with multimorbidity and the impact of socioeconomic factors on results | Contant | 2019 | Quantitative randomised controlled trial | 4 | Yes | Yes | Yes | Yes | No |
| Chronic Care Model for the Management of Patients with Heart Failure in Primary Care | Francesconi | 2019 | Quantitative not randomised | 2 | Can't tell | Yes | Can't tell | Yes | Can't tell |
| Efficacy of a self-management education programme on patients with type 2 diabetes in primary care: A randomised controlled trial | Moreno | 2019 | Quantitative randomised controlled trial | 4 | Yes | No | Yes | Yes | Yes |
| Effects of non-medical health coaching on multimorbid patients in primary care: a difference-in-differences analysis | Shah | 2019 | Quantitative descriptive | 4 | Yes | No | Yes | Yes | Yes |
| A Diabetes Education Model in Primary Care: Provider and Staff Perspectives | Siminerio | 2019 | Qualitative | 4 | Yes | Yes | Yes | No | Yes |
| Effectiveness of Managing Diabetes During Ramadan Conversation Map intervention: A difference-in-differences (self-comparison) design. | Srulovici | 2019 | Quantitative not randomised | 3 | No | Yes | No | Yes | Yes |
| Improving self-management of people with type 2 diabetes in the first years after diagnosis: Development and pilot of a theory-based interactive group intervention | Van Puffelen | 2019 | Mixed methods | 1 | Yes | No | No | No | Can't tell |
| A Coordinated Population Health Approach to Diabetes Education in Primary Care | Zupa | 2019 | Quantitative not randomised | 3 | Yes | No | Yes | No | Yes |
| Activating primary care COPD patients with multi-morbidity through tailored self-management support | Ansari | 2020 | Quantitative not randomised | 2 | No | Yes | No | No | Yes |
| To take charge of one's life - group-based education for patients with type 2 diabetes in primary care - a lifeworld approach | Kjellsdotter | 2020 | Qualitative | 4 | Yes | Yes | Yes | Yes | No |
| Going mobile with primary care: smartphone telemedicine for asthma management in young urban adults (TEAMS) | Mammen | 2020 | Mixed methods | 3 | No | Yes | Yes | No | Yes |
| Improving Glycemic Control in African Americans With Diabetes and Mild Cognitive Impairment | Rovner | 2020 | Quantitative randomised controlled trial | 4 | Yes | Yes | No | Yes | Yes |
| Applying the Chronic Care Model to Improve Patient Activation at a Nurse-Managed Student-Run Free Clinic for Medically Underserved People | Saude | 2020 | Quantitative not randomised | 2 | No | Yes | Can't tell | No | Yes |
| Impact of diabetes education teams in primary care on processes of care indicators | Vitale | 2020 | Quantitative not randomised | 4 | No | Yes | Yes | Yes | Yes |
| Lay Health Coaching to Increase Appropriate Inhaler Use in COPD: A Randomized Controlled Trial | Willard-GRace | 2020 | Quantitative randomised controlled trial | 3 | Yes | No | No | Yes | Yes |
| The effect of structured diabetes self-management education on type 2 diabetes patients attending a Primary Health Center in Kuwait | Alibrahim | 2020 | Quantitative not randomised | 4 | Yes | Yes | Can't tell | YEs | Yes |
| General Behavioral Engagement and Changes in Clinical and Cognitive Outcomes of Patients with Type 2 Diabetes Using the Time2Focus Mobile App for Diabetes Education: Pilot Evaluation | Batch | 2021 | Quantitative not randomised | 2 | No | Yes | No | No | Yes |
| Effectiveness of a Multicomponent Intervention in Primary Care That Addresses Patients with Diabetes Mellitus with Two or More Unhealthy Habits, Such as Diet, Physical Activity or Smoking: Multicenter Randomized Cluster Trial (EIRA Study) | Represas Carrera | 2021 | Quantitative randomised controlled trial | 3 | Yes | Yes | No | Yes | No |
| Latinos understanding the need for adherence in diabetes (LUNA-D): a randomized controlled trial of an integrated team-based care intervention among Latinos with diabetes | Talavera | 2021 | Quantitative randomised controlled trial | 3 | Yes | YEs | Can't tell | Yes | Can't tell |
| The Effectiveness of Telenursing for Self-Management Education on Cardiometabolic Conditions: A Pilot Project on a Remote Island of Osakikamijima, Japan | Moriyama | 2021 | Quantitative not randomised | 3 | Yes | Yes | No | No | Yes |
| Knowledge and practice of home blood pressure monitoring 6 months after the risk and assessment management programme: does health literacy matter? | Fu | 2021 | Quantitative randomised controlled trial | 2 | Yes | No | Can't tell | Yes | Can't tell |
| Impact of a Prescription Produce Program on Diabetes and Cardiovascular Risk Outcomes | Veldheer | 2021 | Quantitative not randomised | 4 | Yes | Yes | Can't tell | Yes | Yes |

Can’t tell: was considered as negative in the score calculation.

1. Hong QN, Fàbregues S, Bartlett G, Boardman F, Cargo M, Dagenais P, et al. The Mixed Methods Appraisal Tool (MMAT) version 2018 for information professionals and researchers. Educ Inf. 2018 Dec 18;34(4):285–91.
